# The Multirole of Liposomes in Therapy and Prevention of Infectious Diseases

**DOI:** 10.3389/fimmu.2018.00155

**Published:** 2018-02-05

**Authors:** Roberto Nisini, Noemi Poerio, Sabrina Mariotti, Federica De Santis, Maurizio Fraziano

**Affiliations:** ^1^Dipartimento di Malattie Infettive, Istituto Superiore di Sanità, Rome, Italy; ^2^Dipartimento di Biologia, Università degli Studi di Roma “Tor Vergata”, Rome, Italy

**Keywords:** liposome, infectious disease, therapy, immunotherapy, drug, vaccine, adjuvant, immunomodulation

## Abstract

Liposomes are closed bilayer structures spontaneously formed by hydrated phospholipids that are widely used as efficient delivery systems for drugs or antigens, due to their capability to encapsulate bioactive hydrophilic, amphipathic, and lipophilic molecules into inner water phase or within lipid leaflets. The efficacy of liposomes as drug or antigen carriers has been improved in the last years to ameliorate pharmacokinetics and capacity to release their cargo in selected target organs or cells. Moreover, different formulations and variations in liposome composition have been often proposed to include immunostimulatory molecules, ligands for specific receptors, or stimuli responsive compounds. Intriguingly, independent research has unveiled the capacity of several phospholipids to play critical roles as intracellular messengers in modulating both innate and adaptive immune responses through various mechanisms, including (i) activation of different antimicrobial enzymatic pathways, (ii) driving the fusion–fission events between endosomes with direct consequences to phagosome maturation and/or to antigen presentation pathway, and (iii) modulation of the inflammatory response. These features can be exploited by including selected bioactive phospholipids in the bilayer scaffold of liposomes. This would represent an important step forward since drug or antigen carrying liposomes could be engineered to simultaneously activate different signal transduction pathways and target specific cells or tissues to induce antigen-specific T and/or B cell response. This lipid-based host-directed strategy can provide a focused antimicrobial innate and adaptive immune response against specific pathogens and offer a novel prophylactic or therapeutic option against chronic, recurrent, or drug-resistant infections.

## Introduction

**Figure 1:**
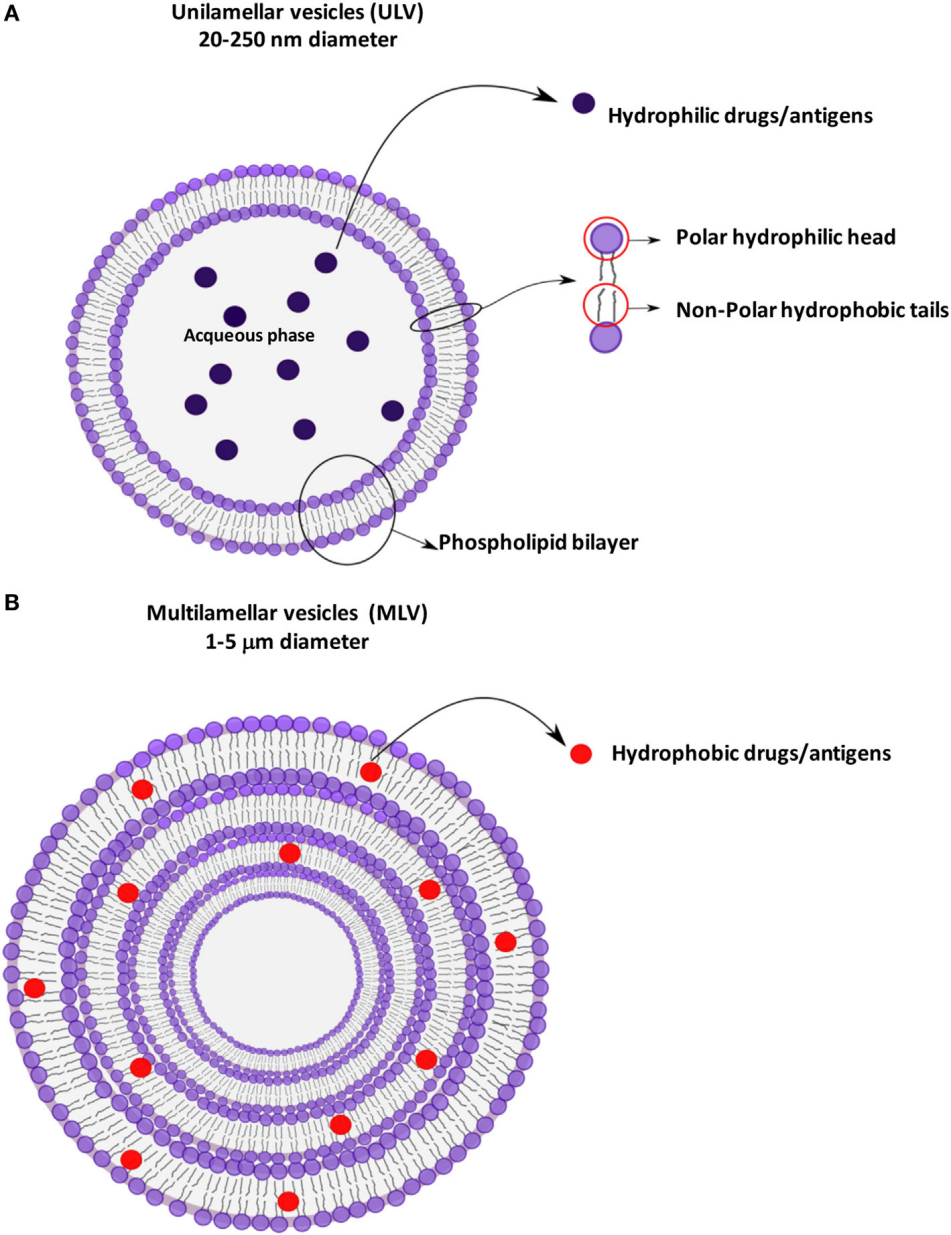
Schematic representation of liposomes showing the different antigen encapsulation propensity: unilamellar **(A)** and multilamellar **(B)** vesicles.

Liposomes are small artificial spherical vesicles constituted by one or more phospholipid bilayers with the polar groups of phospholipids oriented to the inner and outer aqueous phase. Such a structure explains the high propensity of liposomes to be encapsulated with hydrophilic, hydrophobic, and amphiphilic drugs within the inner aqueous compartment, the lipid bilayers, and at their interfaces, respectively (1). Liposomes can be classified as unilamellar (ULV) or multilamellar (MLV) vesicles (2), depending on the structure of the bilayer (Figure 1). ULV are characterized by the presence of a single-lipid bilayer of 20–250 nm diameter, which enclose a large aqueous core and are ideally suited for the encapsulation of hydrophilic drugs/antigens (2). Conversely, MLV are characterized by the presence of two or more concentric lipid bilayers of 1–5 μm diameter that preferentially entrap lipid soluble molecules (2). Liposomes are extensively used as carriers for active molecules in cosmetic and pharmaceutical industries as well as in food and farm industries, where liposomes encapsulation has also been studied to entrap unstable substances such as antioxidants, flavors, and antimicrobials. The phospholipid protective shield forms a barrier, which is normally resistant to the action of enzymes, pH, and free radicals within the body, thus protecting the cargo from degradation until the release occurs at the target cell, organ, or system. Because of the high biocompatibility, biodegradability, low toxicity, and capability to encapsulate hydrophilic and hydrophobic compounds (2), liposomes represent the most successful drug carrier system known to date. Many liposome formulations for the treatment of cancer, fungal, and viral infection as well as pain management are available in clinical use, and many other formulations are being tested in different phases of clinical trials (3). From their first description in 1960s, many liposomes have been produced with distinctive characteristics, which strictly depend on the nature of lipid components, on their possible chemical modifications, and on their surface charge. In particular, early conventional liposomes were mainly constituted by natural phospholipids such as phosphatidylcholine (PC), sphyngomielin, and monosyaloganglioside (4). However, this formulation was subjected to several critical issues, such as the instability in plasma and short blood circulation half-life, due to their interaction with high- and low-density lipoproteins that resulted in the rapid release of the encapsulated drug into the plasma, and the efficient liposome uptake and removal from circulation by the macrophage system. Moreover, in most cases, negatively charged liposomes have a shorter half-life in the blood than neutral liposomes, and positively charged liposomes are toxic and quickly removed from circulation (5). The addition of cholesterol (Chol) in liposome formulation reduced the permeability of lipid bilayer, increased the liposome stability, and reduced the rapid release of encapsulated bioactive compound (6). The size was also shown to be a crucial determinant for circulation half-life of liposomes, as their elimination by phagocytes is directly correlated to the liposome diameter: MLV with diameters ranging from 500 to 5,000 nm are quickly removed by phagocytes, whereas ULV with diameters between 20 and 50 nm show the less propensity to be internalized by macrophages (5), but are characterized by a low volume available for drug entrapment.

**Figure 2:**
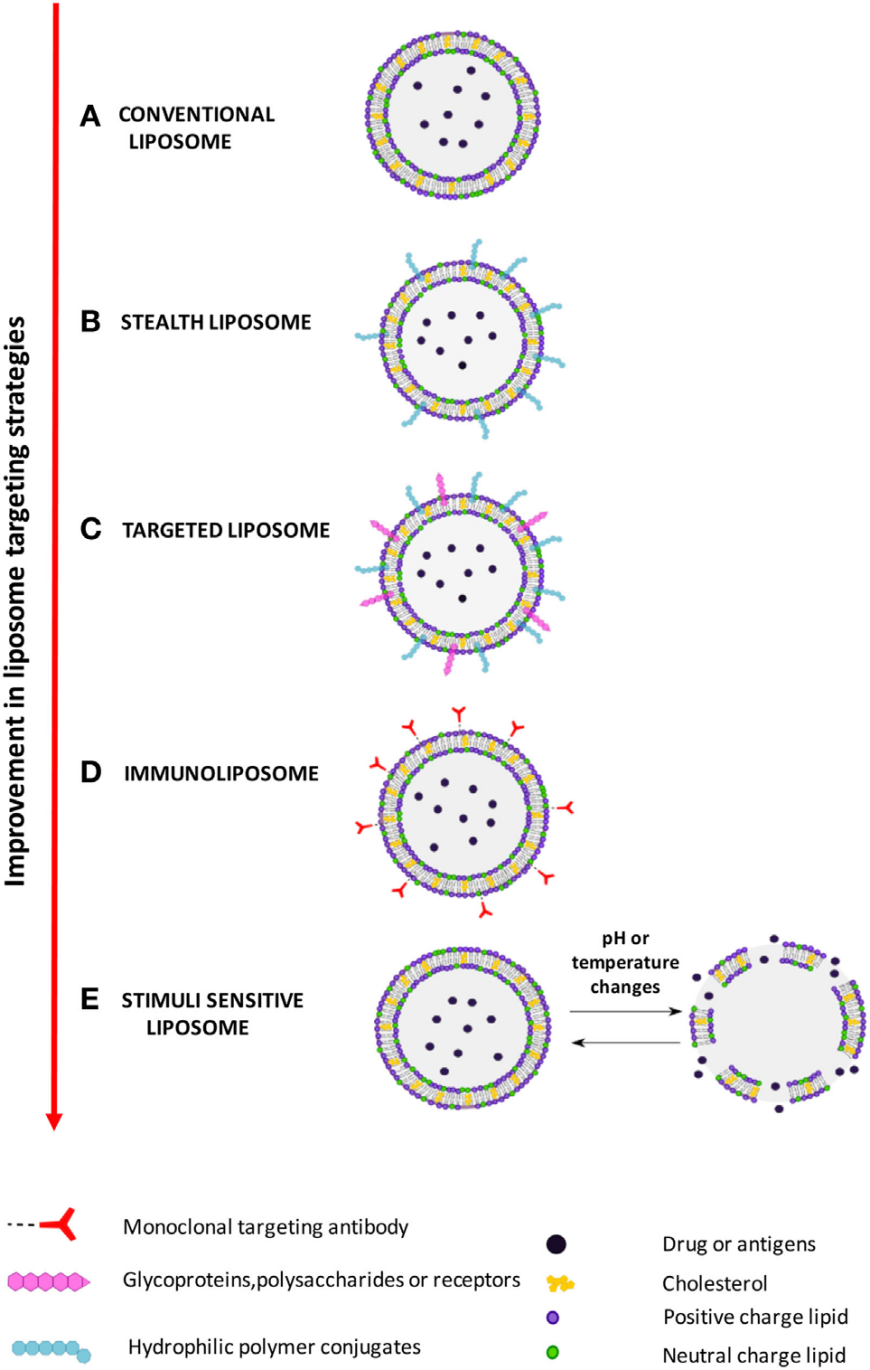
Improvement in liposome targeting strategies. **(A)** Conventional liposomes; **(B)** long circulating stealth liposomes with membrane functionalized with hydrophilic polymeric conjugates, such as polyethylene glycol (PEG), to avoid elimination by reticuloendothelial system; **(C)** targeted liposomes with membranes functionalized with glycoproteins, polysaccharides, or ligands of cell/tissue-specific receptors; **(D)** immunoliposomes, a specific form of targeted liposomes, with membrane functionalized with antibodies or antibody fragments; **(E)** stimuli responsive (pH or temperature) liposomes, which release the encapsulate drug following exposure to acid pH or heating.

Improvement of liposome technology involved the generation of stealth liposome, targeted liposomes, immunoliposomes, and stimuli responsive liposomes (Figure 2). The stealth strategy is based on the possibility to coat the external liposome surface with biocompatible hydrophilic polymer conjugates, such as polyethylene glycol (PEG), chitosan, and others, which reduces immunogenicity and macrophage uptake. Stealth liposome technology makes liposome capable to escape phagocytosis, to reduce toxicity due to the presence of charged lipids, and to increase blood circulation half-life. However, an important limitation of stealth liposomes is their large biodistribution in tissues, as encapsulated bioactive molecules cannot be selectively delivered to target cells (7). Targeted liposomes where therefore designed to counterbalance the large body distribution of stealth liposomes. Targeted liposomes are characterized by the presence of membranes functionalized with glycoproteins, polysaccharides, or ligands for specific receptors that determine the preferential accumulation of liposomes in selected tissues, so that the liposome drug/antigen cargo can be preferentially released in predetermined target cells or organs (7). A further strategy to deliver entrapped drug/antigen in the desired tissue/cell is represented by immunoliposomes, which are functionalized by antibodies or antibody fragments (8), and stimuli responsive liposomes. Examples of stimuli responsive liposomes are the pH-sensitive liposomes (9), which undergo conformational and chemical changes in response to acid pH, and temperature sensitive liposomes (10), which keep their cargo encapsulated at body temperature, but discharge it upon local heating.

In addition to the delivery of drugs, liposomes may also be used for many other purposes through the modification of their composition and cargo. In vaccinology, for example, liposomes can be formulated with the inclusion of antigens (lipids, nucleic acids, proteins, and peptides), and/or with pathogen-associated molecular patterns (PAMPs), which confer adjuvant properties aimed at modulating the inflammatory microenvironment where T lymphocyte priming occurs (11, 12). At the moment, a number of liposome formulations are in clinical trials as adjuvant for prophylactic as well as therapeutic vaccines against malaria, influenza, tuberculosis (TB), human immunodeficiency virus (HIV), and dengue fever (13), whereas Cervarix^®^, Inflexal^®^, and Epaxal^®^ are commercially available liposome vaccines against infection by human papilloma virus (HPV), influenza virus, and hepatitis A virus, respectively. Finally, lipids are important second messengers in the regulation of molecular pathways associated with phagosome maturation and activation of antimicrobial responses (14). Thus, the possibility exists to deliver selected lipid second messengers into infected cells through targeted liposomes to activate antimicrobial molecular pathways against specific bacterial pathogens (15).

This review summarizes the recent developments in liposome technology aimed at the generation of novel prophylactic as well as therapeutic tools to control infectious diseases.

## Liposomes as Carrier for Drugs

The fate of drugs administered to a living organism varies according to several variables, including distribution, metabolism, and excretion. After a drug enters the systemic circulation, it is not distributed uniformly to the body’s tissues due to differences in organ blood perfusion, tissue affinity, local pH, and permeability of cell membrane. Thus, a significant amount of a given drug does not reach the target cell or organ. The consequence of the uneven distribution is a reduced concentration of drug reaching the target cells and the interaction of the drug with bystander targets. Increasing the dose of the drug helps reaching the therapeutic concentration at the target level and also increases the effects of the drug on other cells, concurring to amplify side or adverse events of drug administration. The ratio between the blood concentration at which the drug is effective and the concentration at which the drug becomes toxic is defined therapeutic index (TI): the larger is the TI, the safer is the drug. It has to be further stressed that the ultimate target of drugs is generally an intracellular reaction site and the interaction of drugs with the cellular/bacterial lipid membrane is expected to occur at some point, affecting, sometimes severely, drug bioavailability, and efficacy (16). In the absence of specific mechanisms of intracellular transportation, the chemical nature of the drug dictates the level of entrance into cells. An interesting example is given by the different intracellular accumulation rates observed for four structurally similar quinolones: ciprofloxacin, levofloxacin, garenoxacin, and moxifloxacin (17). Quinolones were shown to exert a condensing effect on the membrane bilayer and the condensing effect differed among the quinolones: even small differences in their chemical structure were shown to influence their lipophilicity, the consequent capacity to cross cell membranes and to accumulate intracellularly, and the eventual antimicrobial effect (18). Thus, the search of innovative approaches to increase drug transport across the cell membrane or to improve the permeability of small molecule, peptide, and/or protein–drugs to access the cytoplasm is a hot field in pharmacology (19).

Within a few years after the first description of “swollen phospholipid systems” by Bangham and colleagues (20), various enclosed phospholipid structures consisting of single bilayers, initially termed “banghaosomes” and then “liposomes” (21), were described (22). The observation that liposomes can entrap drugs made these nanoparticles an interesting tool exploitable as new drug delivery systems (23, 24). The observation that the anticancer drug cytosine arabinoside was successfully delivered by liposomes to tumor cells with a significant increase in the survival times of mice affected by L1210 leukemia (25) was one of the first demonstrations of the suitability of liposomes as drug delivery system. Murine L1210 leukemia became “model system” for testing the therapeutic outcomes of anticancer drugs delivered by a large variety of liposome with different characteristics (26). Another advantage that became evident using liposome-entrapped drugs was the reduction of drug toxicity: drugs entrapped in liposomes are not bioavailable until they are released in target organs. Amphotericin B (AmB) and doxorubicin entrapped in liposomes (26) were extensively studied and the pioneering *in vitro* and *in vivo* studies ultimately led to the first clinical trials of liposomal drugs (27). However, the first tested “large ULV” for drug delivery showed their limits and many efforts have been made to design liposomes able to target specific cells and favor drug release for an optimized release rate of entrapped drug from the liposomes. For the optimal therapeutic activity, drugs must be released from liposomes to the disease site, where they should become bioavailable at a concentration within their therapeutic window for a sufficient time (26). One of the first obstacles that appeared clear when using large ULV was the rapid clearance of liposomes operated by the phagocyte system in the liver and spleen. Several approaches were tested, ranging from variation in liposome dimension to treatment of liposomes with serum albumin or variation in bilayer composition and Chol content (26). A significant step forward in the field of drug delivered by liposomes was made thanks to the observation that attaching PEG to proteins causes an increase of their circulation half-life (28). When the procedure was applied to the liposome system, it was evident that grafting of PEG to the liposome surface resulted in diminished clearance of liposomes by macrophages (29) and, the PEG-liposomes (stealth liposomes), unlike previously tested ULV, were shown to have dose-independent pharmacokinetics (30). The improvements in the therapeutic outcomes of stealth liposomes in comparison with conventional ULV for a variety of therapeutics was demonstrated in different animal models of disease (26), and then in the humans, where the long circulation half-life of a doxorubicin entrapped in PEG–liposomes was confirmed. Shortly thereafter, doxorubicin entrapped in stealth liposomes was used in the first clinical trial for the treatment of Kaposi’s sarcoma in HIV patients (31). The persistence of stealth liposomes in the circulation facilitated their accumulation in highly vascularized sites, such as tumors or inflammatory sites. However, it appeared clear that both the efficacy and the TI would increase if drug-bearing liposomes could be selectively addressed to target cells.

Active targeting could be accomplished by coupling targeting ligands to the surface of liposomes, such as proteins, peptides, carbohydrates, or monoclonal antibodies or their fragments (fragment antigen-binding and single-chain variable fragment) (32). Thus, targeted liposomes can be selectively taken up by cells that overexpress the receptor for the moiety, so improving therapeutic outcomes by increasing efficacy and reducing off-target toxicity (32). Among the different moieties that can be covalently or non-covalently attached to the liposome surface, antibodies and antibody fragments are the most widely employed, producing immunoliposomes (33). Spragg and coworkers demonstrated that E-selectin-targeted immunoliposomes for doxorubicin delivery mediated marked cytotoxicity when incubated with activated human umbilical vein endothelial cells (HUVECs) that express E-selectin, but not when incubated with non-activated HUVECs (34). Following these promising results, methods for antibody coupling to liposomes have been developed (33) and different antibodies or fragments have been attached to liposomes, particularly through reactions with maleimide. Examples include immunoliposomes targeted to soluble *Leishmania* antigens, EFGR for glioma, endoglin (CD105), fibroblast activation protein, and HER2 for breast cancer, among others (35–38).

In addition to the possible systemic use, i.e., intravenous administration (39), liposomes have been designed for aerosol administration (40) or intradermal administration due to their lipophilic properties suitable for skin penetration (41). The functionalization of liposomes makes it possible to design drug delivery systems suitable for the treatment of different diseases according to their pathogenesis and localization. Among other diseases, liposomes have been primarily and deeply studied for cancer treatment. The narrow TI of antitumor drugs, i.e., the narrow window between their effective doses and those at which they produce adverse toxic effects, makes liposomes as ideal carriers for this type of drugs (42). Indeed, the majority of liposome-delivered drugs available for clinical use (Table 1) and in advanced phase III studies to date belongs to this category (3).

**Table 1:** Liposome-delivered drugs in clinical use for the treatment of tumor.

Commercial name	Drug	Liposome composition	Indications
Doxil^®^	Doxorubicin	PEGylated liposome	Advanced ovarian cancer, multiple myeloma and human immunodeficiency virus (HIV)-associated Kaposi’s sarcoma
Myocet^®^	Doxorubicin	Nonpegylated liposome	Breast cancer (43)
DaunoXome^®^	Daunorubicin	Small size liposome	HIV-associated Kaposi’s sarcoma (44)
Depocyt^®^	Cytarabine/Ara-C	Multivesicular particles with a granular structure: DepoFoam™	Neoplastic meningitis (45)
Mepact^®^	Mifamurtide: immunostimulant muramyltripeptide	Multilamellar vesicles	High-grade, resectable, non-metastatic bone tumors combined with postoperative combination chemotherapy in children (46)
Marqibo^®^	Vincristine	Optisomes: sphingomyelin/cholesterol liposome	Adult patients with Philadelphia chromosome-negative acute lymphoblastic leukemia (47)
Onivyde™	Irinotecan	Multilamellar vesicles	Metastatic adenocarcinoma of the pancreas (48)

The property of encapsulation of drugs in liposomes to enhance the TI of various agents was the reason for the first use of anti-infectious drugs in severely ill patients with renal failure. To date, the only drug delivered by liposomes approved for clinical use in the field of infectious disease is AmB, but many studies are ongoing aimed at taking advantage of this versatile platform to treat patients with a variety of different infectious diseases. Three formulations of AmB for parenteral use were made available differing for liposome formulation and indications. Abelcet^®^ was developed in 1995 for the treatment of invasive fungal infections refractory to conventional AmB desoxycholate therapy or when renal impairment or unacceptable toxicity precludes the use of conventional AmB (3). Abelcet^®^ has a 1:1 drug:lipid molar ratio with a drug concentration ranging from 25 to about 50% molar (49). Ambisome^®^ was approved in 1997 for the treatment of severe fungal infections including leishmaniasis, aspergillosis, blastomycosis, coccidioidomycosis in febrile, neutropenic patients, and a severe form of meningitis in individuals infected with HIV (3). Ambisome^®^ is also indicated for the treatment of invasive systemic infections caused by *Aspergillus* sp., *Candida* sp., or *Cryptococcus* sp. in renal impaired patients or in patients who cannot tolerate therapy with free AmB (50). Amphotec^®^ is a freeze-dried lipid-based formulation of AmB. The sodium salt of cholesteryl sulfate forms a thermodynamically stable colloidal complex with AmB at a 1:1 drug-to-lipid molar ratio (3).

In the field of parasitic infections, several studies were performed to test the efficacy of liposome-encapsulated drugs. In the treatment of leishmaniosis, an interesting approach is based on the use of dinitroanilines, because of their specific binding to parasite but not human tubulins. However, their low water solubility and instability have blocked their development as antiparasitic agents (51). Encapsulation of drug by dimyristoyl PC- and dimyristoyl phosphatidylglycerol-based liposomes overcome those limitations and allowed to efficiently deliver the drugs, reaching a therapeutic advantage as demonstrated in animal models (51). Another drug under test for liposome delivery is buparvaquone, an extensively studied drug for the treatment of visceral leishmaniosis. da Costa-Silva et al. reported that buparvaquone, which has an immunomodulatory effect in host cells, when entrapped in phosphatidylserine (PS) exposing-liposomes, as a delivery approach to macrophage, is highly effective at low doses at eliminating *Leishmania infantum* parasites in a hamster model (52).

In the fight against malaria, the majority of drugs under development are lipophilic and characterized by poor plasma solubility and large biodistribution volumes with low accumulation in red blood cells (RBC). As a consequence, these drugs have shown limited therapeutic activity against intra-erythrocyte *plasmodia* (53). Rajendran et al. developed lipid formulations of soy-PC Chol containing either stearylamine (SA) or phosphatidic acid (PA) and different densities of distearoyl phosphatidylethanolamine-methoxy-PEG 2000 as a delivery system to test the antimalarial activity of monensin. Monensin entrapped in such liposome formulations was tested both in *in vitro* systems of *Plasmodium falciparum* cultures and in *in vivo* murine models of *Plasmodium berghei* infection and found to be more effective than free monensin given at comparable doses (54). The trans platinum–chloroquine diphosphate dichloride was recently successfully tested after its encapsulation into PEGylated neutral and cationic liposomes to fight parasites resistant to traditional drugs and proposed as a new therapeutic tool against malaria (55). Preliminary assays conducted in 1987 using passively loaded chloroquine into RBC-targeted immunoliposomes resulted in significant *P. berghei* growth inhibition in mice, when compared with administration of the free compound (56). More recently, the immunoliposome strategy against malaria was tested taking advantage of a monoclonal antibody specific for a 52-kDa RBC surface antigen characteristic of the murine erythroid lineage and specifically expressed from the early proerythroblast to mature RBC stages. These immunoliposomes loaded with two novel antimalarial lipophilic drug candidates in the mouse model of *Plasmodium yoelii* increased the TI and efficacy of the used drug (53). Liposomes exposing PS-specific peptide may represent a further strategy to target *plasmodia* infected RBC in apoptosis (eryptosis:erythrocyte apoptosis) that have been tested in malaria treatment, with promising results (57). Experimental malaria may also represent a model to study the combined effect of anti-infective drugs and anti-inflammatory compounds aimed to limit immunopathogenic reactions in critical anatomic sites, such as the brain. Guo et al. tested liposome-encapsulated β-methasone hemisuccinate in a murine model of experimental cerebral malaria and found that this anti-inflammatory liposome-delivered drug prolonged the survival time of infected animals, permitting the administration of antimalarial drug before the development of severe anemia (58). In addition to malaria, liposomes, as part of nanopharmaceuticals, are promising tools under investigation for the treatment of several other parasitic diseases including schistosomiasis (59, 60) and acanthamoebiasis (61), which are grouped under the term of neglected diseases (62).

In 2016, it was estimated that there were nine million new TB cases in the world and around half a million of them were caused by multidrug-resistant (MDR) *Mycobacterium tuberculosis* (Mtb) strains (Global tuberculosis Report 2017. http://www.who.int/tb/publications/global_report/en/ Health Organization, Geneva, Switzerland). The research is focused in developing new antimicrobials, but the need to reach a sufficiently high drug concentration inside the lung macrophages and the appearance of MDR and extensively drug-resistant (XDR) Mtb strains represent two major obstacles to reach this goal (63, 64). A possible strategy to the development of new anti-TB therapeutics is based on the administration of drugs contained in specific nanodevices (62), which, preferentially targeting macrophages, allows the use of lower drug dosage and the reduction of undesirable side effects as well as the limitation of Mtb resistance mechanisms. In this line, an interesting contribution to the development of innovative anti-TB therapeutic strategies is the inhalation therapy with liposomes carrying anti-TB drugs. The inhaled drug is expected to be rather effective in the overt presence of bacteria as in smear-positive cases of TB in which the bronchial tree may be directly connected with the cavitary lesions, where Mtb rapidly multiplies. Liposomes delivering anti-TB drugs may offer several advantages over dry powder inhalable formulations of anti-TB drug (65, 66) or other aerosol administrable nanoparticles (67), such as the increase of drug half-life and the possibility to target the intracellular pathogen (68). However, difficulties may arise to efficiently entrap some of the drugs required for TB treatment into liposomes. Justo and Moraes showed that passive encapsulation of isoniazid and pyrazinamide in liposomes during dry lipid film hydration at initial drug-to-lipid molar ratios of 13.3 can be achieved with efficiencies of 2.5 and of 2.2%, respectively, equivalent to final drug-to-lipid molar ratios of 0.33 and 0.29 (69). Moreover, they were also able to incorporate ethionamide during the dry lipid film production step with a 42% trapping efficiency, equivalent to a low final drug-to-lipid molar ratio of 0.04 (69). Justo and Moraes were unable to efficiently incorporate rifampicin and streptomycin in the vesicles under the conditions tested. In contrast, Zaru et al. succeeded in entrap rifampicin in liposomes made of phospholipon 90^®^, soy lecithin, and Chol using the film hydration method followed by procedures of freeze dry (70). A deeper deposition of rifampicin entrapped in these liposomes in comparison with rifampicin suspension was found in lung airways of rats after nebulization of a nose-only exposure chamber, encouraging the use of liposomes in aerosol therapy of TB (71). An additional aspect to be faced is the stability of liposome-entrapped drugs. Stability may be enhanced by the use of pro-liposomes, i.e., free flowing granular products composed of drug and phospholipid precursors, which on hydration lead to liposomes (72, 73) that have a higher stability upon reconstitution. A new single-step and fast spray drying technique for pro-liposome powder preparation was developed (74, 75) using a systematic method known as “Quality by Design” (71): a systematic approach to develop drug products that includes evaluation of formulation parameters to achieve defined final product quality (76). The method was used to prepare rifapentine-loaded pro-liposomes, which resulted in an average size of 578 nm and an efficiency of encapsulation around 70% (71). The pro-liposome with a drug/hydrogenated soy-PC ratio of 1:2 and SA as a charge-inducing agent (74, 75) are promising formulations and represent possible new tools to approach therapy of infectious diseases based on the optimization of the delivery of old drugs. However, liposomes may be also considered for the delivery of new developed drugs to reduce or delay the appearance of drug resistance.

Liposome-entrapped drugs or immunomodulating agents are being developed for the treatment of many other diseases (see some examples in Table 2). However, clinical trials based on innovative liposome strategies should take into account also possible side effects that these drug formulations may have, particularly when given intravenously, and the fulfillment of several regulatory landscape. Both FDA and EMA have developed specific guidelines to be fulfilled in clinical trials with reference to future novel liposomal products (77, 78).

**Table 2:** Possible applications of liposome-encapsulated drug.

Disease/field	Drug	Reference
Glaucoma	Dorzolamide	(79)
Uveoretinitis	Imiximab	(80)
Age-related macular degeneration	Verteporfina	(3)

Pulmonary hypertension	Phosphodiesterase 5 inhibitors	(81)
Post ischemia	Streptokinasis	(82)

Pain management	Diacylglycerol lipase-beta inhibitors	(83)
Anesthesia	Bupivacaine	(84, 85)

Skin disorders	Various drug	(41, 86, 87)

Autoimmune diseases	Infliximab, other immunosuppressants	(80, 88, 89)

Others	Nucleic acid	(87)
	siRNA	(90)

## Liposomes as “Direct” Enhancers of Innate Antimicrobial Immune Response

**Figure 3:**
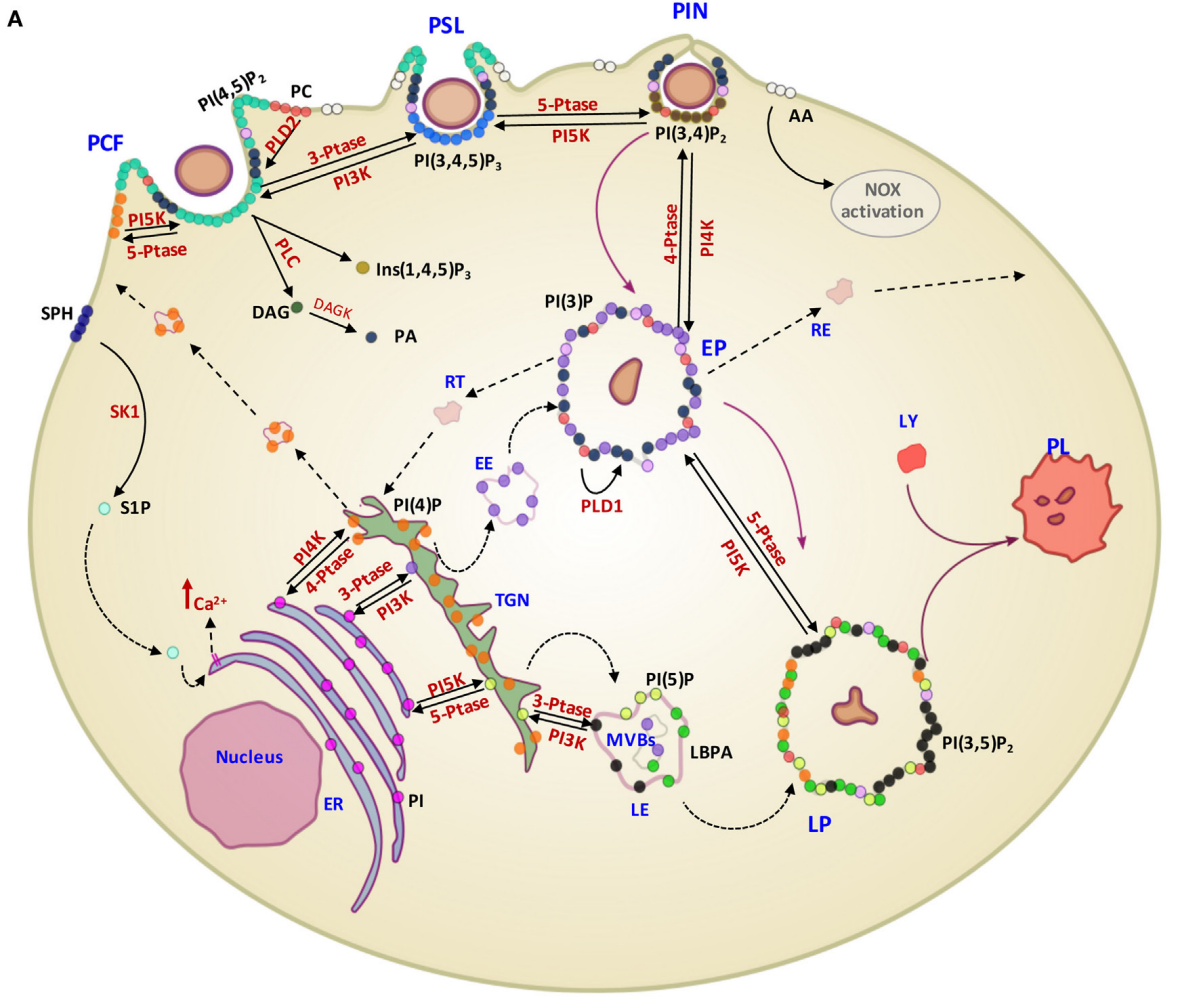

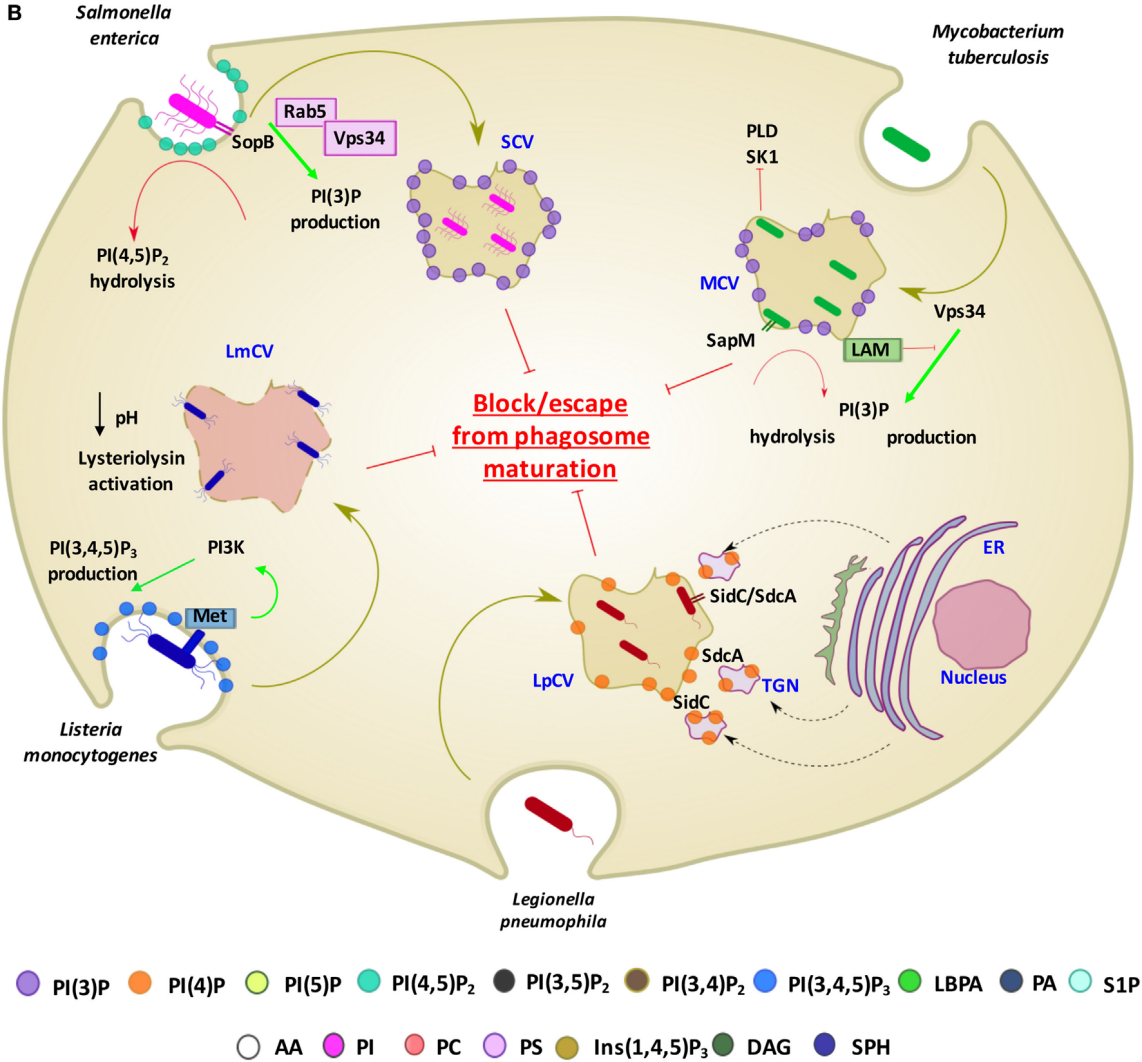
Contribution of different lipid second messengers to phagocytosis from phagosome formation to PL maturation and examples of interference by pathogens. **(A)** The first event which occur during particulate engulfment is the PCF and the rapid accumulation of PI(4,5)P_2_ levels, mediated by PI5K and made possible thanks to the translocation of PI(4)P from Golgi to the membrane. PI(4,5)P_2_ can also be converted in Ins(1,4,5)P_3_ and DAG by the means of PLC and, furthermore, DAG can be metabolized to PA through the DAGK activity. PA is also early generated by the activation of PLD2, which hydrolyzes PC to PA and choline. Moreover, in this stage, there is a rapid generation of S1P, produced by the phosphorylation of sphingosine by the means of SK1, which in turn may activate sphingolipid Ca^2++^-release-mediating protein of endoplasmic reticulum (SCaMPER). Following, at the stage of PSL, PI(4,5)P_2_ is phosphorylated to PI(3,4,5)P_3_ by PI3K, which is converted to PI(3,4)P_2_ by 5-PTase during PIN. EP is characterized by the presence of PI(3)P, resulting by both its translocation from Golgi and the dephosphorylation of PI(3,4)P_2_, mediated by 4-PTase. Although PA is present in the early stage of phagocytosis process, at this stage its conversion starting to PC is mediated by the action of PLD1. LP is characterized by the presence of PI(3,5)P_2_, PI(5)P, and LBPA. In particular, PI(3,5)P_2_ can be generated by the enzymatic activity of both PI5K and PI3K, starting from PI(3)P and PI(5)P, respectively. PI is the precursor of PI(3)P, PI(4)P, and PI(5)P and their phosphorylation is mediated by PIK3, PI4K, and PI5K, respectively, in ER. **(B)** Examples of pathogen interference with host lipid metabolism associated with phagosome maturation. *Salmonella enterica* secretes the PI phosphatases SopB, which has a direct effect on the PI(4,5)P_2_ hydrolysis and an indirect effect on PI(3)P production through the recruitment of Rab5 and its associated enzyme PI3K (Vps34). *Mycobacterium tuberculosis* (Mtb) produces a secretory acid phosphatase (SapM) and LAM, which hydrolyzes PI(3)P and inhibites the generation of PI(3)P, respectively. Moreover, Mtb is able to inhibit SK1 and PLD action. *Lysteria monocytogenes* invades cells by binding to the host cell Met receptor, which leads to activation of PI3Ks and PI(3,4,5)P_3_ production and promotes a partial phagosome maturation resulting in a pH decrease and lysteriolysin activation. *Legionella pneumophila*, sequesters endosomes, enriched in PI(4)P, from TGN through SidC and SdcA bacterial proteins, and hence creating replicating vacuoles for itself. *Abbreviations for lipids*: PI(3)P, PI 3-phosphate; PI(4)P, PI 4-phosphate; PI(5)P, PI 5-phosphate; PI(4,5)P_2_, PI 4,5-bisphosphate; PI(3,4)P_2_, PI 3,4-bisphosphate; PI(3,4,5)P_3_, PI 3,4,5-trisphosphate; LBPA, lysobisphosphatidic acid; PA, phosphatidic acid; AA, arachidonic acid; PI, phosphatidylinositol; PC, phosphatidylcholine; PS, phosphatidylserine; Ins (1,4,5)P_3_, inositol 3,4,5 trisphosphate; DAG, diacylglycerol; SPH, sphingosine; S1P, sphingosine 1-phosphate; LAM, lipoarabinomannan. *Abbreviations for enzymes*: PLD-1, phospholipase D-1; PLD-2, phospholipase D-2; PLC, phospholipase C; DAGK, diacylglycerol kinase; SK1, sphingosine kinase 1; PI3K, phosphoinositide 3-kinase; PI4K, PI 4-kinase; PI5K, phosphoinositide 5 kinase; 3-PTase, PI 3-phosphatase; 4-PTase, PI 4-phosphatase; 5-PTase, PI 5-phosphatase; *Other abbreviations*: PCF, phagocytic cup formation; PSL, phagosome sealing; PIN, phagosome internalization; EP, early phagosome; LP, late phagosome; PL, phagolysosome; LY, lysosome; EE, early endosome; LE, late endosome; MVBs, multivesicular bodies; RE, recycling endosome; RT, retrograde traffic; TGN, trans Golgi network; ER, endoplasmic reticulum; SCV, *Salmonella* containing vacuole; MCV, *Mycobacterium* containing vacuole; LmCV, *Listeria* containing vacuole; LpCV, *Legionella* containing vacuole.

For long time, the function of lipids was believed to be limited to their role as structural components of membranes or principal form of energy storage in cells. However, during the past 30 years, their important role in cell physiology has been disclosed and lipids have been identified as fundamental players in many cellular functions, including, among others, the regulation of signaling, organization of membranes, intracellular vesicle trafficking, and phagocytosis. Particularly interesting is the role of lipids in the physiology and pathology of phagocytosis, which represents a critical step in the effector function of innate immunity. In fact, the phagosome formation and maturation are functions involving coordinated signaling, targeting, and trafficking events largely regulated by lipid moieties (91, 92). In this process, several lipid species accumulate in membrane microdomains, where they associate in signaling transduction platforms (93). In addition, several lipids can (i) promote positive (convex) or negative (concave) membrane curvature, (ii) recruit signal proteins by binding to specific lipid-binding domains (Table 3), and (iii) confer an electrostatic potential on membrane to attract opposite charged key signaling and effector proteins (91–93). Thus, intracellular vesicle trafficking is finely regulated by a topologically and temporally controlled lipid expression whose main role, in the case of phagocytosis, is to promote the fusion/fission events, which starting from phagosomes leads to the generation of a bactericidal phagolysosome (PL) (14). Phagocytosis starts with particle recognition by phagocytic receptors and proceeds with phagocytic cup formation, phagosome sealing (PSL), and internalization (Figure 3A). In particular, one of the first events occurring during phagocytic receptor signaling is a local rapid accumulation of phosphatidylinositol (PI) 4,5-bis phosphate [PI(4,5)P_2_] (94), mediated by PI 4-phosphate 5-kinases (PIP5Ks), whose activity both regulates phospholipase D (PLD) and is regulated by PA, the product of PLD (95). Few minutes after accumulation, PI(4,5)P_2_ is rapidly degraded by phospholipase Cγ (PLCγ) or phosphorylated, by type I phosphoinositide 3-kinase (PI3K), to PI(3,4,5)P_3_ (96), whose formation is required for pseudopod extension and phagosome formation (97). The rise of PI(3,4,5)P_3_ at sites of phagocytic receptor engagement promotes the recruitment of PLCγ through its pleckstrin homology (PH) domain (98), with the consequent hydrolysis of PI(4,5)P_2_ to diacylglycerol (DAG), which in turn can be converted in PA by DAG kinases (99). PA, PI(4,5)P_2_, and PI(3,4,5)P_3_ represent the most important lipid second messengers involved in the early phases of phagocytosis. These lipids contribute to the lateral spreading of phagocyte receptor signaling, which is important to integrate the signals elicited by sparsely phagocytic receptors and to cytoskeletal remodeling. Remodeling culminates with the formation of pseudopods, which surrounding the phagocytic target and sealing at their distal tips, form the phagosome (96). Phosphoinositides are key molecules in modulating cytoskeletal reorganization starting from the early phases of phagocytosis: PI(3,4,5)P_3_ contributes by recruiting WAVE and myosin X to forming phagosomes, while PI(4,5)P_2_ can promote actin polimerization by (i) inducing WASP dependent Arp2/3 activation, (ii) removing capping proteins like profilin from the ends of actin filaments, and (iii) stimulating gelsolin-mediated severing of actin filaments and hence allowing fast growth of barbed ends (91). PI 3-kinase (Vps34) is expressed in early phagosomes (EPs) and causes enrichment in PI 3-phosphate (PI(3)P), which is crucial for EP maturation since it recruits multiple effectors through highly specific interactions with FYVE and phox-homology (PX) domains on target proteins, such as early endosome antigen-1, endosomal sorting complex required for transport (ESCRT)-0, sorting nexins, and p40phox of NADPH oxidase (100, 101). In EPs, also PA has an important role because it contributes to the assembly and activation of NADPH oxidase by interacting directly with p47phox and by recruiting and activating a number of protein and lipid kinases, including Fgr, the type I PIP5KI, and type 1 sphingosine kinase (14, 102). During the transition from early to late phagosome (LP) stage, PI(3)P is then lost as a consequence of hydrolysis catalyzed by a phosphoinositide lipid 3-phosphatases and PI 4-phosphate [PI(4)P] is rapidly acquired as a result of the enrichment in PI(4)P kinase 2A and such acquisition is associated with fusion of autophagosomes with lysosomes (LYs) (103). LP stage is also characterized by the presence of PI 5-phosphate [PI(5)P] and PI 3,5-bisphosphate [PI(3,5)P_2_]. In particular, PI(3,5)P_2_ can be generated by the enzymatic activity of both PI3K and phosphoinositide 5 kinase (PI5K), starting from PI(3)P and PI(5)P, respectively, and takes part in endolysosome morphology, acidification, and trafficking (104). PI(5)P can be directly produced from PI by the means of the PI5K or by dephosphorylation of PI(3,5)P_2_ by myotubularin 3-phosphatases and seems to be involved in actin remodeling and in protein sorting in endosomal compartment (105).

**Table 3:** Lipid-binding domains and protein interaction of the most representative bioactive lipids involved in phagocytosis process.

Lipid	Binding domains	Reference
PI(3)P	FYVE	(106)
	PX	(107)

PI(4)P	ENTH	(108)
	PH	(109)

PI(5)P	PHD	(110)

PI(4,5)P_2_	ENTH	(111)
	ANTH	(112)
	PH	(113)
	C2	(114)
	FERM	(115)
	PTB	(116)

PI(3,5)P_2_	ENTH	(117)
	GRAM	(118)

PI(3,4)P_2_	PX	(119)
	PH	(107)

PI(3,4,5)P_3_	PX	(119)
	PH	(120)
	PTB	(121)

PA	PH	(122)
	PX	(123)
	C2	(124)

**Protein interactions**		

LBPA	Alix binding	(125)

S1P	SCaMPER	(126)

AA	NOX-2 activation	(127)

*PI, phosphatidylinositol; PI(3)P, PI 3-phosphate; PI(4)P, PI 4-phosphate; PI(5)P, PI 5-phosphate; PI(4,5)P_2_, PI 4,5-bisphosphate; PI(3,5)P_2_, PI 3,5-bisphosphate; PI(3,4)P_2_, PI 3,4-bisphosphate; PI(3,4,5)P_3_, PI 3,4,5-trisphosphate; PA, phosphatidic acid; LBPA, lysobisphosphatidic acid; S1P, sphingosine 1-phosphate; AA, arachidonic acid; PH, pleckstrin homology; PX, phox-homology*.

Phospholipids may also contribute to the membrane surface charge. Changes in the content of anionic phospholipids are accompanied by marked alterations in the surface charge of the membrane of nascent phagosome, where proteins that associate with the membrane by electrostatic interactions may relocate (128). For example, the dislocation of phosphoinositides [prevalently PI(4,5)P_2_ and PI(3,4,5)P_3_] causes the modification of surface charge observed after PSL, and the different charge facilitates the dissociation of target proteins causing the termination of signaling and cytoskeleton assembly (92). Similarly, during phagosome maturation, the cytosolic leaflet of phagosomes remains negatively charged and this is prevalently due to accumulation of PS (129). Such a negative charge serves to target proteins with polycationic clusters or polybasic domains and seems to be an important determinant in the distribution of both synaptotagmins, which control membrane fusion events (130), and small GTPases of the Rab and Rho superfamilies, which are critical for both phagosome formation and maturation (131). Thus, the level of anionic phospholipids, including PS and phosphoinositide species, may provide an electrostatic switch to control protein recruitment and vesicle trafficking.

In addition to activation of different signaling pathways, phospholipids can promote phagosome maturation process by inducing positive or negative membrane curvatures. For example, PA is a cone-shaped (type II) lipid that induces negative (concave) curvature of membranes (132), whereas lysobisphosphatidic acid, an inverted cone-shaped fusogenic lipid, forces positive (convex) membrane curvature (125). The resulting changes in membrane curvature activate specific curvature sensors, which are protein motifs or domains, such as amphipathic lipid-packing sensor motifs and BAR domains, which act as geometry sensing tools (133). These sensors play important roles in membrane trafficking and remodeling, by regulating membrane protein concentration, enzyme activation, lipid composition, and vesicle fusion events (134).

The translocation of PS on the outer leaflet of plasma membrane represents one of the early events of apoptosis, occurring in both infected and uninfected apoptotic cells, and represents an “eat-me” signal for macrophages (135–138). The functional consequence of a PS-dependent recognition and phagocytosis of apoptotic bodies (efferocytosis) by macrophages is the release of TGF-β, IL-10, and the inhibition of the production of pro-inflammatory cytokines, such as TNF-α and IL-1β (139). These features have been previously reported as a possible therapeutic strategy to reduce immunopathologic responses (136, 140). However, if phagocytosis involves apoptotic bodies derived from cells expressing PAMPs, as in the case of apoptosis mediated by an infectious agent, phagocytosis causes activation of antigen-presenting cells (APCs) and release of IL-6, TGF-β, and IL-23, leading to a preferential activation of pro-inflammatory Th17 cells (141). Moreover, apoptotic bodies from macrophages infected by *Mycobacterium bovis* bacille Calmette–Guerin (BCG) have been reported to promote antigen cross-presentation and CD8^+^ T cell activation as well as an *in vivo* increased protection against experimental TB in mice (142). Coherently, emulsified PS has been described as a simple and efficient carrier to deliver antigenic peptide epitopes to professional APCs to induce both helper and cytotoxic T cell response *in vivo* (143).

Considering the crucial role of lipids in the activation of antimicrobial response, many intracellular bacterial pathogens evolved strategies aimed at interfering, at different levels, with lipid metabolism to generate specific pathogen-containing-vacuoles permitting their intracellular survival (14) (Figure 3B). *Salmonella enterica* exploits type III secretion system to secrete the PI phosphatases SopB, a phosphoinositide phosphatases able to dephosphorylate PI(4,5)P_2_. Decreased levels of PI(4,5)P_2_ reduce the surface charge of the *Salmonella* containing vacuole (SCV) and promote membrane fission by reorganizing the actin cytoskeleton during bacterial internalization (144). SopB also recruits the RAB GTPase RAB5 protein and the RAB5 effector Vps34, a PI3K that accumulates PI(3)P on the SCV surface and interferes with the maturation to next LP stage (144). *Listeria monocytogenes* invades host cells by recognition of Met receptor by bacterial internalin B (InlB), which leads to the PI3K activation and consequent enhancement of PI(3,4,5)P_3_ levels. Such increase in PI(3,4,5)P_3_ promotes the fusion of early endosomes with the bacterial vacuole, allowing partial maturation of phagosome before lysis of the phagosome and escape into cytoplasm due to the pH-dependent activation of listeriolysin (145). *Legionella pneumophila*, through SidC and SdcA proteins, anchors to PI(4)P expressing endosomes from trans Golgi network to form a replicative niche for the pathogen (146). Mtb, as well as BCG, interferes with PL maturation (15, 136, 147) by depleting the phagosome of PI(3)P and hence arresting the vacuole in an immature form (148). The mycobacterial secretory acid phosphatase (SapM) acts in concert with the mycobacterial lipoarabinomannan (LAM), a phosphatydilinositol analog, to avoid the formation of PI(3)P by hydrolysis of the phosphate group (149) and by inhibiting the activity of type III PI3K Vps34 (150), respectively. Finally, an inhibition in host PLD activation was observed during early phases of interaction between Mtb and macrophage (151). This inhibition was not due to differences in protein expression, suggesting a metabolic control of the enzyme. Interestingly, an upstream inhibition of sphingosine-kinase exerted by Mtb was also reported which may be responsible of the reduced calcium mobilization (152) and the consequent inhibition of Ca^++^-dependent PLD activation (153) observed during *in vitro* Mtb infection.

Given the well-defined role of certain lipids in the physiology of phagocytosis, the possibility to modulate the phagosome maturation process by the use of bioactive lipids has been suggested as a strategy to increase the efficacy of innate bactericidal mechanisms. In this context, Anes et al. showed that selected bioactive lipids, such as arachidonic acid, ceramide, sphingosine, sphingomyelin, and sphingosine 1-phosphate (S1P), activate phagosome actin assembly and maturation resulting in killing of pathogenic mycobacteria in murine macrophages (154). Moreover, S1P and its analog lysophosphatidic acid (LPA) were both described to activate PLD-mediated and PL maturation-dependent mycobactericidal response in human macrophages (155, 156) and in type II alveolar epithelial cells (157) in *in vitro* models of Mtb infection, highlighting the role of PA in the activation of antimycobacterial response. Interestingly, and of high translational value, both lysophospholipids promoted antimicrobial response, resulting in a significant intracellular killing of endogenous mycobacteria, in bronchoalveolar lavage (BAL) cells from pulmonary TB patients (155, 158). Finally, the intranasal administration of S1P or LPA in murine models of primary Mtb infection significantly reduced pulmonary mycobacterial burden and histophatology (156, 159, 160). On these grounds, the targeted delivery of several bioactive lipids into macrophages to increase their innate anti-bacterial mechanisms could be useful for the treatment of Mtb infection. However, such a delivery is challenging, due to lipid pharmacokinetic properties and to the difficulties to target specific cells. The possibility to incorporate lipid second messengers in Janus-faced liposomes may represent a strategy to overcome these limitations (15, 136). The main feature of these liposomes is the asymmetric distribution of phospholipids within the liposome membrane, with inner leaflet expressing bioactive lipids involved in phagosome maturation, and the outer surface expressing PS, representing an “eat-me” signal for macrophages. PA delivered by this strategy allowed to enhance PL maturation-dependent (myco)bactericidal response of macrophages and BAL cells from TB patients as well as from patients affected by MDR bacterial pneumonia (15, 136). Notably, both PA and PI(5)P delivered by Janus-faced liposomes were able to enhance antimicrobial response against *Pseudomonas aeruginosa* in an *in vitro* cellular model of cystic fibrosis by using macrophages expressing a pharmacologically inhibited or a naturally mutated cystic fibrosis transmembrane conductance regulator (15), which are characterized by impaired phagosome maturation (161). These experimental evidences suggest the possibility to use liposomes to deliver bioactive lipids to enhance phagosome maturation-dependent antimicrobial response and to restore the PL maturation pathway, which can be corrupted by specific pathogens (15).

## Liposomes as “Indirect” Enhancers of Adaptive Antimicrobial Response

Vaccines represent a great contribution of medical sciences to the reduction of the deaths caused by infectious diseases. The majority of currently available vaccines induce neutralizing or opsonizing pathogen-specific antibody responses to protect against infectious diseases. However, a number of diseases are caused by pathogens that are not controlled by humoral responses and novel vaccine formulations aimed at generating protective pathogen-specific immune responses are under testing or development. According to the pathogen virulence factors or mechanisms of pathogenesis, novel vaccines are being designed to enhance either cellular immune responses, with prevalence of cytotoxic T cells, or polarization of specific T cell subpopulations, or aimed at increasing the response at mucosal or systemic sites. One of the strategies currently under development is based on the use of recombinant antigens (subunit vaccines) in association with adjuvants able to modulate/polarize the developing immune response. Recombinant antigens show a higher safety profile and lower reactogenicity, but are characterized by a reduced immunogenicity in comparison with inactivated or attenuated whole cell vaccines. As a consequence, adjuvants are generally included to vaccine formulations to provide the necessary inflammatory microenvronment required for the activation of innate immunity cells and the following priming and expansion of naïve T cells for the development of appropriate adaptive immune response. Thus, adjuvants are generally defined as substances that (i) facilitate a depot effect and deliver the antigen in proximity of APC, (ii) generate an inflammatory microenvironment necessary for the activation of APC, and (iii) induce the secretion of specific cytokine patterns by APC for the differentiation of naïve T cells into different CD4^+^ and/or CD8^+^ T cell subpopulations.

In addition to their suitability as drug carriers, liposomes represent an extraordinary tool for the devise of innovative vaccines, as they can be designed for the antigen delivery (see chapter 5) and/or for their possible adjuvancy function (162). In fact, several liposome adjuvants have been licensed for human use and others are being evaluated in clinical trials (Table 4). However, many other formulations are possible and have been or could be tested for efficacy and safety. In this regard, different liposome adjuvant formulations can be produced with different properties according to the (i) lipid composition, (ii) liposome size, (iii) liposome charge, and (iv) inclusion of function modifiers such as PAMPs.

**Table 4:** Examples of liposome adjuvants vaccines against infectious disease in market or tested in clinical trials.

Target	Immuno modulators	Lipid formulation	Antigen	Phase	Clinical trials.gov identifier
HAV	HA + NA	DOPC:DOPE	Inactivated HAV	Licensed (Epaxal^®^)	
Human papilloma virus	MPLA + aluminum hydroxide (AS04)	n.d.	L1	Licensed (Cervarix^®^)	
Influenza virus	–	DOPC:DOPE	HA	Licensed (Inflexal^®^)	
Influenza virus	–	CCS/C (“VaxiSome”)	HA	II	NCT00915187
*Mycobacterium tuberculosis*	TDB (CAF01)	DDA	Ag85b + ESAT6	I	NCT00922363
*Plasmodium Falciparum*	MPLA + QS21 (AS01)	n.d.	RTS,S	III	NCT00872963
*P. falciparum*	MPLA + QS21 (AS01)	n.d.	FMP012	I	NCT02174978
*P. falciparum*	MPLA + QS21 (AS01)	n.d.	RH5.1	I/IIa	NCT02927145
*P. falciparum*	EPA/AS01	n.d.	Pfs25M + Pfs230D1	I	NCT02942277

*This compilation was generated with data from ClinicalTrials.gov*.

*DOPC, dioleoylPC; DOPE, dioleoly-sn-glycero-phophoethanolamine; DDA, dimethyldioleoylammonium; HA, hemaglutinin; NA, neuroaminidase; TDB, trehalose6,6-dibehenate; MPLA, monophosphoryl lipid A; QS21, triterpene glucoside compound derived from the *Quillaja saponaria* tree; CCS, *N*-palmityol-derythro-sphingosyl-Carbamoyl-Spermine; CCS/C, *N*-palmityol-derythro-sphingosyl-Carbamoyl-Spermine/Cholesterol; EPA, ExoProtein A (a detoxified form of exotoxin A from *Pseudomonas aeruginosa*); n.d., not determined; CFA, cationic adjuvant formulation; HAV, hepatitis A virus*.

### Lipid Composition

The choice of liposomal lipid composition (in terms of hydrocarbon length, unsaturation, charge, and headgroup species of the lipids) influences the physico-chemical features of the liposomes, such as the lipid bilayer fluidity, which can in turn influence immune response. For instance, the degree of saturation or the length of fatty acids may affect the strength of the van der Waals forces among neighboring chains. Thus, phospholipids with longer saturated hydrocarbon chains show higher tendencies to interact each other and to form rigidly ordered bilayer structures than shorter and/or unsaturated hydrocarbon chains, which form liposomes with fluid and disordered bilayers (163). Membrane liposome fluidity in turn affects antigen presentation as it has been previously shown that fluid disordered phase liposomes promoted MHC class I presentation pathway to a higher degree than the solid ordered liposomes (164). Furthermore, in *in vivo* studies, solid ordered liposomes prepared using dimethyldioleoylammonium (DDA) induced 100-fold higher Th1 response than the fluid liposome counterpart prepared using the unsaturated analog DDA bromide (165). Inclusion of Chol is also known to influence bilayer fluidity and is commonly incorporated within liposomes as it can enhance liposome stability. The impact of Chol on adjuvancy is actually controversial, with some studies showing improvements and others reduction of immune response (166). Cationic adjuvant formulation (CAF)01 is actually a commercially available liposome adjuvant formulation that is composed of DDA stabilized with trehalose 6,6-dibehenate (TDB), a glycolipid synthetic variant of mycobacterial cord factor. TDB activates macrophages and dendritic cells (DCs) *via* Syk–Card9–Bcl10–Malt1 signaling pathway and when administrated in combination with a Mtb subunit vaccine induced a robust and combined antigen-specific Th1 and Th17 responses and partial protection against Mtb challenge (165, 167).

### Liposome Size

The dimension of liposomes can impact adjuvant efficacy and several studies have shown that Th1 or Th2 response can be evoked by using particles with different diameter (168). In this context, a significantly higher Th1 response, as measured in terms of IL-12-dependent IFN-γ secretion and serum IgG2a production, has been reported following vaccination with large vesicles (≥225 nm diameter), whereas the same antigen encapsulated in small liposomes (≤155 nm diameter) induced a prevalent Th2 response as determined in terms of IgG1 and increased lymph node (LN) IL-5 production (169). Such a different outcome can reflect differences in particle trafficking to LNs: small particles (20–200 nm) freely traffics to the draining LNs where they are taken up by LN resident DCs, whereas larger vesicles (500–2,000 nm) are internalized by tissue-resident DC (170). Different antigen presentation pathway has also been observed following internalization by phagocytes: antigen encapsulated in large (560 nm) vesicles localized in early immature phagosomes, where class II antigen-processing pathway could be intercepted for recognition by CD4^+^ T cells, while antigen encapsulated in small (155 nm) vesicles were rapidly transferred to late endosomes/lysosomes, with a consequent reduced class II-restricted Ag-presenting efficiency (171).

### Liposome Charge

Liposome charge may have critical effects on the adjuvant properties of liposomes. Cationic liposomes may interact with the negatively charged mucosal surfaces and exibit increased mucoadesion, leading to reduced clearance rate, prolonged exposure time of the antigen at the mucosal surface (depot effect), enhanced endocytosis of liposomes by APC (13, 172–175), and increased cell-mediated immune response (173) when compared with neutral liposomes, which tended to induce antibody immune response (176). However, anionic liposomes may also modulate immune response in the context of novel formulations of liposomal adjuvants. As above described, PS is expressed on the outer membrane leaflet of apoptotic bodies and represents an “eat-me” signal for macrophages and DC, which express specific PS receptors. Phagocytes that internalize apoptotic bodies through PS became polarized toward a tolerogenic response (177). However, when the recognition occurs in the presence of selected PAMPs a Th17-differentiating cytokine profile is produced (170) and a similarly efficient class II- and class I-restricted antigen presentation is induced (143).

### Function Modifiers

The procedures of liposome preparations make possible to include within the liposome structure different function modifiers molecules, such as PAMPs. The addition of function modifiers molecules may permit to design APC-targeted liposomes able to modulate the effector function of APC, including the profile of secreted cytokine that, in turn, can induce the differentiation of diverse T helper cell subpopulations (178). For example, the immunization of mice with liposomes containing ovalbumin (OVA) and oligodeoxynucleotide (ODN) with CpG motifs (ligand for TLR-9) induced a Th1-type immune response, with enhanced production of IFN-γ and of IgG2a, whereas the same antigen encapsulated with Pam3CSK4 (a TLR-2 ligand consisting of tri-palmitoyl-*S*-glyceryl cysteine lipopeptide with a pentapeptide SKKKK) induced increased levels of IgG1, suggesting a Th2-oriented immune response (179, 180). Another example is given by the lipophilic muramyldipeptide (MDP) analogs MDP phosphatidylethanolamine as well as the MDP glyceroldipalmitate that induced higher antibody titers, Th1 response, and IFN-γ levels if added to liposomal formulations containing hepatitis B surface antigen (HBsAg) (181). Thus, the use of different PAMPs can modulate the immune response against the same antigen, in dependence on the targeted TLR.

Monophosphoryl lipid A (MPLA) is a safe and potent liposome adjuvant used with many candidate antigens for new vaccines in the fight against several types of cancer and anti-infective vaccines such as malaria and HIV-1 (182). For example, RTS,S (a particulate antigen consisting of hepatitis B particles coexpressing epitopes derived from *P. falciparum* circumsporozoite protein) induced an *in vivo* cytotoxic T-lymphocyte response and a dose-dependent enhancement of the specific IgG levels when entrapped in liposomes containing MPLA and not when encapsulated in liposomes missing MPLA (183). Moreover, a recent paper demonstrated that the *P. falciparum* Protein-013 can induce a potent and sterilizing immune response when formulated with small ULV containing a synthetic MPLA derivative (3D-PHAD^®^) and the QS-21 (184). Similarly, Nagill and Kaur encapsulated the 78-kDa antigen of *Leishmania donovani* with MPLA, resulting in decreased parasite burden after challenge in immunized mice (185).

Korsholm et al. described a novel CD8^+^ T-cell-inducing adjuvant, CAF09, consisting of DDA-liposomes stabilized with monomycoloyl glycerol-1 (a synthetic analog of a mycobacterial cell wall lipid and a potent stimulator of human DCs) and combined with the TLR3 ligand Poly(I:C) (186). CAF09 was used to test immunogenicity of the model antigen OVA as well as several antigens of Mtb (TB10.3, H56), HIV (Gag p24), HPV (E7) in mice and resulted in a high frequency of antigen-specific CD8^+^ T cells (186). In the mouse model of subcutaneous HPV-16 E7-expressing TC-1 skin tumor, immunization with the E7 antigen and CAF09 as adjuvant significantly reduced the growth of the tumor (186). Another widely studied liposome carrier is the cationic liposome–DNA complex (CLDC) that is prepared by mixing liposomes and ODN with CpG motifs, which activate TLR9. Bernstein and colleagues demonstrated that CLDC increased the immune response against simian immunodeficiency virus induced by vaccines. The immunization of rhesus macaques with CLDC induced a potent virus-specific B- and T-cell response in comparison to control animals (12, 187). Moreover, in mice expressing hepatitis B virus, the CLDC was tested as adjuvant for HBsAg. Whereas HBsAg induces only a B-cell immune response, the viral antigen formulated together with CLDC elicited both T- and B-cell responses (12, 188).

## Liposomes as Antigen Carrier in Innovative Vaccine Preparations

Allison and Gregoriadis were the first to describe the capacity of liposomes to elicit immune responses against associated or incorporated antigens (12, 189). Water-soluble compounds (peptides, proteins, carbohydrates, nucleic acids, and haptens), depending on the chemical properties, can be linked to the surface of liposomes, by stable chemical bond or by adsorption, or can be trapped inside the aqueous inner space, whereas lipophilic compounds (antigens, lipopeptides, linker molecules, and adjuvants) can be inserted into the lipid bilayer (12, 190). Surface-exposed antigens are available for and can stimulate B-cell for antibody production, while both surface-exposed and encapsulated antigens, which require intracellular liposome disruption to be accessible, may induce T-cell responses. Liposome-encapsulated protein antigens have been frequently used and several studies well documented the MHC class-I presentation and induction of cytotoxic T lymphocytes (CTLs) by antigens formulated inside liposomes. H-2 antigens entrapped in egg lecithin plus Chol (30% w/w) liposomes induced a potent anti-H-2 CTL allo-response (191). Human LS174T colon tumor cell membranes encapsulated into liposomes (PC/Chol/PA 7:2:1) elicit *in vitro* specific primary and secondary xenogeneic immune responses in murine splenocytes (192). Induction of antiviral immunity by liposome-encapsulated peptides was tested by Ludewig and colleagues that showed the high immunogenicity of peptides derived from the glycoprotein of the lymphocytic choriomeningitis virus when administered intradermally in mice (193). In addition, these authors used the liposome to prime a strong MHC class I-restricted T-cell response specific for 10 diverse epitopes of hepatitis C virus (HCV) (12, 194). In the fight against Ebola Zaire (EBO-Z) virus, the protective efficacy of liposome-encapsulated [L(EV)] irradiated EBO-Z, containing all of the native EBO-Z proteins was evaluated (195). Mice immunized intravenously with L(EV) and challenged with a uniformly lethal mouse-adapted variant of EBO-Z were totally protected from illness and death compared with mice vaccinated intravenously with the irradiated virus non-encapsulated that presented a 55% of survival (195). Liposomes were also formulated with the pH-sensitive 3-methyl-glutarylated hyperbranched poly(glycidol) (MGlu-HPG) and used to entrap OVA. Such MGlu-HPG formulations elicited a strong T cell activation that was inhibited using blocking antibodies against MHC class-I/MHC class-II molecules, suggesting the involvement of MHC class I- and II-restricted antigen presentation (12, 196). Ding and colleagues developed the so-called RAFTsomes, a liposome incorporating membrane microdomains of APCs with enriched epitope/MHC complexes. OVA epitope loaded RAFTsome immunization gave high anti-OVA IgG1 levels and the immunized mice were protected from OVA-expressing EG.7 tumor cell inoculation challenge (197).

Mucosal surfaces are the main entry site for most environmental antigens and the mucosal immunity plays a critical role in preventing the initial infection of many pathogens. Several studies are focused on figuring out the ability of liposomes to act as efficient mucosal antigen delivery system. A potential mucosal carrier formulation has been developed by Gupta and Vyas that encapsulated HBsAg in liposomes coupled with *Ulex europaeus* agglutinin 1, a lectin isolated from *U. europaeus* seeds. After oral immunization, lectinized liposomes were predominantly targeted to M cells on intestinal Peyer’s patches, inducing a strong anti-HBsAg IgG response in serum, anti-HBsAg IgA in various mucosal fluids, and cytokine levels in the spleen homogenates (198). Figueiredo and colleagues described another mucosal formulation composed of *Streptococcus equi* antigens encapsulated inside PC/Chol/SA liposomes or inside chitosan nanoparticles. Mice immunized intranasally with both delivery systems developed mucosal, humoral, and cellular immune responses, but higher secretory IgA levels in the lung were observed following vaccination with chitosan nanoparticles, due to their enhanced mucoadhesive properties (12, 199). Wang and colleagues encapsulated the OVA in a galactosylated liposome carrier in which a galactosyl lipid (galactose conjugated covalently with 1,2-didodecanoyl-sn-glycero-3-phosphoethanolamine) was incorporated into a liposomal bilayer. OVA-encapsulated targeted galactosylated liposome elicited in the nasal and lung wash fluid a significantly higher OVA-specific secretory IgA titers and serum IgG antibody levels than control mice (200). Traditional phosphodiester liposomes are not stable and could be easily degraded in the gastrointestinal tract. Zhang and colleagues described the archaeosomes, a novel oral delivery system based on the polar lipid fraction E isolated from *Sulfolobus acidocaldarius*. The archaeosomes had significant higher stability in simulated gastric and intestinal fluids after oral administration and induced a strong serum IgG as well as mucosal IgA immune response. Moreover, such delivery system elicited antigen-specific MHC class I-restricted T cell proliferation (201).

Liposomes are versatile tool to encapsulate and deliver a wide range of molecules, not only proteins but also glycolipid antigens. Kallert et al. demonstrated that purified mycobacterial LAM can be efficiently delivered into CD1^+^ APC *via* liposomes and this delivery system induces robust LAM-specific Th1-biased CD1-restricted T-cell responses in primary human cells (202). The authors used liposomes consisted of egg-PC, Chol, and stearylated octaarginine and proved that octaarginine, a cell-penetrating peptide consisting of eight positively charged arginines, increases the glycolipid antigen accumulation into lipid-APCs. The efficacy of octaarginine-containing liposomes was also tested in *in vivo* models of immunization using both BCG primed guinea pigs (203), where these liposomes induced a delayed type hypersensitivity reaction in the skin and in rhesus macaques, using glucose monomycolate (GMM) as antigen, where a CD1-restricted Th1-skewed immune response was observed (202, 204). In a recent study, GMM-containing liposomes were decorated with a high-affinity glycan ligand of the sialic acid-binding Ig-like lectin-7, a siglec receptor expressed on DC that mediates rapid endocytosis and transport of its cargo to LYs (205). This elegant targeting platform leads to a robust GMM-specific CD1-restricted activation of T cells. One way to ensure rapid and specific liposome uptake by leukocyte subsets is decoration of liposomes with antibodies against a cell subset-specific antigen. Klauber et al. reported a liposomal drug delivery system for robust and specific targeting of monocytes and DCs consisting of sterically stabilized liposomes with surface-conjugated antibodies against C-type lectin “Dendritic Cell Immunoreceptor” (206). Using this method, they were able to activate the targeted cells and improve the ability of the agonist to induce secretion of key anticancer cytokines (IL-12p70, IFN-γ, and IFN-α).

Typically, exogenous antigens residing in proteolytic PLs are directed to MHC class II-expressing compartments (MIIC) where peptides resulting from antigen proteolysis are loaded on the neoformed class II molecules to be expressed on cell membrane. Thus, bioactive lipids like PI(3)P, promoting (auto)PL biogenesis, can in turn promote class II antigen presentation pathway (207). Harding and colleagues (208) used liposomes composed of dioleoyl PC/dioleoyl PS (DOPC/DOPS) or dioleoyl phosphatidylethanolamine/palmitoyl homocysteine (DOPE/PHC) at 4:1 molar ratios with four different encapsulated protein antigens (OVA, murine hemoglobin, bovine ribonuclease, or hen egg lysozyme) and proved that macrophages efficiently processed the encapsulated Ag. These liposomes were able to sequester their contents from potential endosomal processing events and release them only after delivery to LYs for efficient MHC class II presentation (208).

The antigen presentation pathway leading to the loading of exogenous antigens on MHC class I molecules is called cross-presentation. Cross-presentation can occur *via* a vacuolar and a cytosolic pathway. The vacuolar pathway bypasses the cytosolic steps and TAP- or proteasome-sensitivity, is dependent on lysosomal proteases, and invokes a peptide exchange step for reloading of endocytosed MHC-I complexes, which are then rerouted directly back to the plasma membrane in a Brefeldin A-insensitive manner (209). The cytosolic route, considered to be the most important, is TAP and proteasome dependent and allows the export of the antigen into the cytosol through the acquisition of the traslocon SEC61 on antigen-containing endosomes from the endoplasmic reticulum (ER)-associated degradation pathway (210). Although no specific lipid second messengers promoting cross-presentation pathway have been identified so far, the involvement of specific membrane contact sites between ER and phagosomes (211) suggests that specific signal lipids can be involved. The permanence of the antigen in a non-proteolytic compartment represents a condition promoting cross-presentation and explains why DC, whose phagosomes show reduced acidity and limited proteolysis, have better cross-presentation capability than macrophages. In this context, cationic liposomes, but not anionic liposomes, that increase the lysosomal pH in DCs and reduce antigen degradation, promote antigen cross-presentation and CD8^+^ T-cell cross-priming (212, 213). As an alternative strategy to promote antigen delivery to cytoplasm and make it available for class I antigen presentation, Miura et al. developed liposomes carrying OVA modified with KALA peptide, an α-elical cationic peptide derived from the sequence of the N-terminal segment of the hemagglutinin (HA)-2 subunit of the influenza virus HA, which is involved in the fusion of the viral envelope with the cell membrane (214), and show membrane destabilizing properties (215). MHC class I presentation pathway can also be favored using pH-sensitive liposomes. For example, Reddy et al. have shown that OVA entrapped in pH-sensitive liposomes (DOPE/1,2-dioleoyl-sn-glycero-3-succinate 1:1 and DOPC/PS/Chol 5:2:3) allowed MHC class I presentation of OVA peptides by mouse thymoma cells, which were lysed by OVA-specific CD8^+^ T lymphocytes (216).

Liposomes can be designed to carry antigens within the acqueous phase, encapsulated inside the lipid bilayer, or exposed onto the liposome surface and this can result in different outcomes of the immune response. Investigation of HA adsorption versus encapsulation and coencapsulation of ODN with CpG motifs in 3β-[*N*-(*N*′,*N*′-dimethylaminoethane)-carbamoyl] Chol (DC-chol) liposomes demonstrated that encapsulated HA was less immunogenic than adsorbed HA (217). Moreover, Takagi and colleagues immunized human-HLA-transgenic mice with liposomes exposing several epitope-peptides derived from HCV in their external layer and observed that 1 mouse- and 3 human MHC-restricted peptides protected and conferred long-term memory to vaccinated animals (218). Immunization of HLA-A*0201-transgenic mice with liposomes conjugated with peptides designed according to the sequence of highly conserved antigens of influenza viruses and HLA-A*0201 binder, induced antigen-specific CD8^+^ T-cells, and mediated protection of mice challenged intranasally with the influenza viruses H1N1 or H3N2 (219). Similarly, when the peptide nucleoprotein (NP)366–374, designed according to the sequence of the NP of influenza H3N2 virus, was coupled with liposomes and used to immunize mice induced peptide-specific CD8^+^ T-cells and the replication of influenza H3N2 virus was successfully suppressed in the lung of challenged mice (220). Naito and colleagues reported that OVA chemically coupled with the surface of liposome *via* amino groups using glutaraldehyde induced Ag-specific IgG but not IgE Ab production (221), showing that liposomes can be exploited to develop vaccines devoid of allergenic potential. Moreover, liposomes showed different capacities to induce class I or class II presentation of the delivered antigen depending on the mobility of their membranes. Taneichi et al. reported that OVA coupled with liposome constituted by unsaturated fatty acid was presented to both CD4^+^ and CD8^+^ T lymphocytes, while OVA conjugated to liposomes constituted by saturated fatty acid were not presented to CD8^+^ T lymphocytes (222). The *in vivo* induction of CTL and the eradication of E.G7 tumor in mice confirmed the capacity to induce cross-presentation by Ag-conjugated liposomes prepared including unsaturated fatty acid in the bilayer (222). Masek and colleagues used small Ni-chelating liposomes exposing on their surface the *Candida albicans* His-tagged antigen hsp (hsp90-CA) with the MDP derivative C18-*O*-6-norAbuMDP as adjuvant. These liposomes were phagocytosed by human DCs *in vitro* and *in vivo* in mice, where induced antigen-specific Th1 and Th2 responses without side effects (223). At least in theory, the covalent attachment of protein antigens to nanocarriers could modify protein structure and mask epitopes, altering the antibody response. Watson and colleagues tested this hypothesis using metal chelation *via* nitrilotriacetic acid (NTA) to attach antigens to liposomes instead of covalent linkage. OVA and a peptide derived from the membrane-proximal region of HIV-1 gp41 (N-MPR) were attached *via* NTA or covalent linkage. Covalently attached N-MPR or OVA elicited stronger antibody responses than NTA-anchored antigens excluding the possible masking effect of covalent binding (224). Finally, antigen modification may also impact the response against haptens as reported by a study by Matyas et al., where the authors conjugated haptenic compounds to protein carriers and embed them in the outer surface of MPLA-containing liposomes. Four synthetic opiate haptens were conjugated to carrier proteins and induced high levels of specific antibodies to synthetic heroin haptens following *in vivo* immunization that might be useful as a candidate vaccine to heroin and similar opiates (225).

Self-amplifying messenger RNA (mRNA) technology has been developed for *in situ* expression of antigens and represents a new platform for innovative vaccine applications (226) with the advantage that unlike DNA, mRNA-based vaccines do not integrate into chromosomes avoiding the risks of oncogenesis and insertional mutagenesis (227). Liposomes were identified as the more suitable delivery system for non-amplifying or self-amplifying mRNA vaccine, and it was rapidly evident that mRNA encapsulated inside liposomes can activate innate immunity through toll-like and RIG-I-like receptors (228, 229).

Richner and colleagues tested the efficacy of this novel technology in a vaccine against Zika virus using a modified mRNA-encoding prM-E gene that produced virus-like particles with a reduced cross-reactivity with the related dengue virus (230). Two immunizations induced a strong protective humoral response against Zika infection in mouse model and reduced the cross-reactive response against the related virus dengue. For this experiment, liposomes were obtained taking advantage of a method developed by Chen and colleague, consisting of ionizable lipid, DSPC, CHOL, and PEG-lipid (dissolved in ethanol at the molar ratios 50:10:38.5:1.5) combined with a citrate buffer containing mRNA (3:1 = aqueous:ethanol, pH 4.0) using a microfluidizer. The dialyzed and concentrated liposome preparations resulted in 80–100 nm in size and more than 90% encapsulation (231).

The mRNA-based liposomes were also tested to induce humoral responses against influenza A virus in a mouse model. Lipid nanoparticles were engineered to deliver a synthetic, self-amplifying mRNA encoding seasonal influenza HA and tested their immunogenicity in mouse model. This mRNA-based vaccine elicited an immune response comparable to that obtained with the licensed influenza subunit vaccine and all the immunized animals showed HA inhibition and neutralizing antibody titers against the virus 14 days after the second immunization (232). In another study, the immunogenicity of lipid nanoparticles encapsulating a modified non-replicating mRNA encoding influenza H10 HA was tested in *rhesus macaques*. This study showed that anti-HA protective antibody titers and MHC class II-restricted H10-specific T cell response were elicited after two intradermal or intramuscular immunizations. Moreover, this study provided evidences on cells and mechanisms responsible for the efficacy of mRNA vaccinations: following the administration of mRNA–liposomes, DCs, monocytes, and neutrophils were recruited to the site of immunization and draining LNs, but only DCs and monocytes internalized liposomes, produced virus antigens, translating the mRNA, and upregulated costimulatory molecules. This *in vivo* antigen production and activation of APCs lead to the priming of a potent H10-specific MHC class II-restricted T cell response (233). The superiority of liposome encapsulation versus other delivery systems of mRNA was stressed by the group of Bogers and colleagues, who proved the immunogenicity and the safety of cationic liposomes formulated with the mRNA encoding for a clade C envelope glycoprotein of HIV in *rhesus macaques*. This formulation elicited a cellular immune response and a neutralizing antibody response stronger than those elicited by self-amplifying mRNA encapsulated in a viral replicon particle or by a recombinant HIV envelope protein formulated with MF59 adjuvant (234).

## Engineering Multirole Liposomes Able to Deliver Drugs, Antigens, Adjuvants, and/or Lipid Second Messengers as an Additional Tool to Contrast Infectious Diseases

Infectious diseases are the leading causes of morbidity and mortality worldwide and the increasing incidence of treatment-resistant infections has become a major global concern for their epidemic potential (235). In 2016, WHO reported 6 × 10^5^ new cases of TB with resistance to rifampicin, the most effective first-line anti-TB drug, of which 4.9 × 10^5^ had MDR TB and about 9.5% of MDR-TB cases have additional drug resistance and were defined XDR-TB.^1^ Moreover, 2.3 × 10^4^ in US^2^ and 2.5 × 10^4^ deaths in Europe^3^ are attributable each year to infections caused by Gram-positive (*Streptococcus penumoniae, Staphylococcus aureus*), and Gram-negative (*Pseudomonas* spp., *Acinetobacter* spp., and *Enterobacteriaceae*) bacteria with multidrug- or pan-antibiotic resistance. As a consequence, the development of novel antimicrobial agents and/or other immunotherapeutic options represents a global health priority (236). To this regard, several therapeutic strategies, targeting the host rather than the pathogen, have been proposed and are under development as adjunctive treatments, in particular for MDR TB (235). Examples of host-directed therapies are the reinfusion of *in vitro* expanded pathogen-specific autologous T cells, the administration of micronutrients, antimicrobial peptides, or immune modulators and therapeutic vaccines (235).

**Figure 4:**
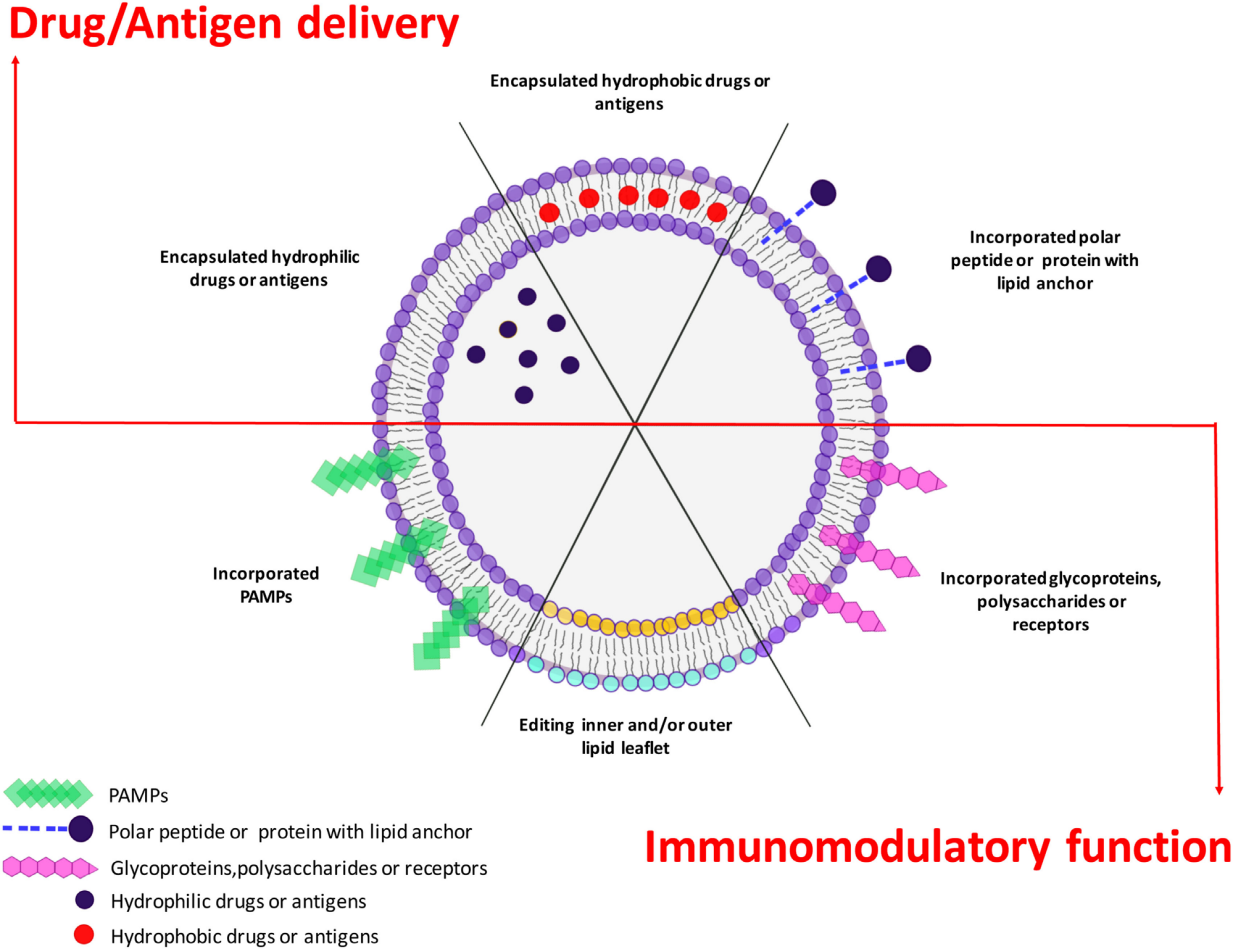
Plasticity of liposome technology. Liposome technology allows combining the selective delivery of drugs or antigens (upper side) with immunomodulatory functions played by incorporated pathogen-associated molecular patterns (PAMPs) or selected bioactive phospholipids (lower side). The simultaneous delivery of antigen/drugs and immunomodulatory molecules makes liposomes a versatile platform to design different therapeutic or prophylactic tools to face pathogen-specific strategies.

Liposomes may offer several advantages in the design of novel pathogen- or host-directed therapies and vaccines, since these nanoparticles can be engineered as delivery system as well as immune modulators. In clinical applications, liposomal drugs have been proven their ability to “passively” accumulate at sites of increased vasculature permeability and for their ability to reduce the side effects of the encapsulated drugs in comparison to free drugs (237). This has resulted in an overall increase in TI, so that liposomal drug delivery has become an established technology platform in some circumstances and has gained considerable clinical acceptance (26). A new frontier in liposome-based approaches against infectious diseases may be envisaged if liposomes are designed as carrier of antigens or drugs in addition to molecules with immunomodulatory functions (Figure 4). For example, antigen-loaded liposome carrying different PAMPs inside or exposed on their outer surface leaflet, would target plasma membrane-associated or intracellular pattern-recognition receptors, respectively, and consequently activate APC to secrete different cytokines, which in turn may drive diverse polarizations of the antigen-specific adaptive immune response (11). It is also possible to engineer asymmetric liposomes that express different lipids at the outer and inner membrane leaflet (15, 136, 238). In this regard, PS may be included in the constitutive lipids of the outer liposome leaflet to target specific cells, like macrophages, whereas lipid second messengers, such as PA or PI(3)P, can be incorporated in the inner leaflet to be delivered to the cell microvesicular system to modulate intracellular endosome trafficking (15, 136, 238). Pathogens may target host lipid metabolism and PL maturation as an intracellular survival strategy and mechanism of immunoevasion (14, 144–146, 148–152, 238–241). The growing evidences for a crucial role played by lipid second messengers in PL biogenesis (91–94, 129) opens new therapeutic possibilities for the reactivation/enhancement of antimicrobial innate immune response with the specific intracellular delivery of lipid second messengers taking advantage of the liposome technology. Since scaling up, stability and regulatory issues are commonly successfully addressed, the clinical availability of novel liposome formulations can be foreseen in the near future. The possibility to enhance/correct specific molecular pathways by lipid second messengers, used for liposome scaffold, may represent a further added value to the plethora of different possibilities offered by liposomes technology and a possible novel host-directed therapy to face the global emergence of antimicrobial resistance.

## Author Contributions

All authors made substantial contributions to the conception or design of the work. NP, SM, and FS contributed to the acquisition, analysis, interpretation of data for the work. NP and FS drew the figures. RN and MF wrote the work. All authors revised critically the work for important intellectual content, gave final approval of the version to be published, and agreed to be accountable for all aspects of the work in ensuring that questions related to the accuracy or integrity of any part of the work are appropriately investigated and resolved.

## Conflict of Interest Statement

All the authors declare no business relationship that might lead to a conflict of interest.

## Footnotes

^1^http://www.who.int/tb/publications/global_report/en/.

^2^https://www.cdc.gov/drugresistance/threat-report-2013/index.html.

^3^http://www.ema.europa.eu/ema/index.jsp?curl=pages/news_and_events/news/2009/11/news_detail_000044.jsp&mid=WC0b01ac058004d5c1.
